# The Association of Medical Officers of Asylums and Hospitals for the Insane

**Published:** 1853-07-01

**Authors:** 


### THE ASSOCIATION OF MEDICAL OFFICERS OF ASYLUMS AND HOSPITALS FOR THE INSANE.

An attempt is now being made to resuscitate a suggestion made last year, at the meeting held at Oxford, in relation to the establishment of a journal in connexion with the Association of Medical Officers of Asylums for the Insane. The editor of this journal is indebted to the courtesy of a friend for a copy of Dr. Bucknill's circular, having reference to this subject. We have a right to ask, why this circular was distributed to other members of the Association, and carefully withheld from ourselves? Again, how was it that the whole plan of the new journal was conceived, and even its editor selected, before the meeting of the Association last year at Oxford? It appeared from the statement of Dr. Williams of the Gloucester Asylum, one of the secretaries of the Association, that he and Dr. Bucknill had been in correspondence with some of the members of the Association on the subject of the journal, prior to the meeting last year, but the editor of this journal received no communication in relation to this matter. Being a member of the Association, the secretary and Dr. Bucknill had no right to exclude us from the council, and as an act of courtesy, if for no other consideration, we should have been apprized of the objects they had in contemplation. We say thus much, because we feel that such a mode of conducting the business of the Association is calculated to seriously diminish its influence. We again say, if it were considered necessary to have a new Psychological Journal to represent the opinions of this

Association, the selection of the editor should have been left to its members. But how is this appointment made? At the meeting at Oxford, Dr. Williams, the secretary, refers to the fact of his having been in communication with the members of the Association in relation to the proposed journal, and then concludes his observations by saying, *that he was happy to inform the members present, that his friend Dr. Bucknill had kindly volunteered to become the editor of the proposed periodical!* A member then asked, who was to defray the expenses of the publication? Dr. Williams in reply said, that the annual subscription of one guinea would entitle the members to a copy of the journal, and *that if the expense exceeded the aggregate amount paid by the members, Dr. Bucknill would defray it out of his own pocket!* Now, we ask, is this a right mode of transacting the business of the Association? Who delegated Dr. Williams, the secretary, with the power of originating a journal to be identified with the Association, and by what authority did he place himself in communication with the members on the subject? Surely the proper course was, to give the members due notice that such a proposition would be made at the next meeting, and if adopted, the Association should have had the power of nominating its own editor. Common courtesy should have dictated the propriety of this course; but to come down to the meeting at Oxford with the plan "cut and dry," and the appointment of editor actually given away, is not a proceeding that will bear the test of serious business-like examination. It may be said, "Oh! you or the members could have objected to the journal, or to the appointment of Dr. Bucknill as its editor." But was it likely that any member of the Association would place himself in a position so personally offensive as to object to Dr. Bucknill's appointment, it having been *previously* arranged by Dr. Williams that *he* was to have the conduct of the journal? We wish it to be clearly understood that in making these remarks we disclaim all idea of questioning Dr. Bucknill's ability to edit a journal of the kind; but we do maintain that the proceedings at Oxford in relation to this matter has placed the Association under a temporary cloud. We understand that few members of any influence or status in that Association have responded favourably to Dr. Bucknill's circular. Many distinguished members have distinctly informed us that they have positively refused to write for it, on the ground that a second psychological journal is not needed. We must confess we do not feel ourselves complimented at the suggestion to establish a periodical of the kind. Our pages have always been open to the communications of the members of the Association, and we have done our utmost to promote its well-being, to advance medico psychological literature, and to support the interest of those connected with the public asylums of this country. Having embarked a capital of some thousand pounds in establishing this journal, and having, since 1848, stood nearly alone in fighting the battle for the British psychologist, it cannot be otherwise than mortifying that those who have never lifted their little finger to assist us, should, in 1853, attempt to injure the property of this journal by starting a rival publication? One word as to the Association itself. For all practical and useful purposes is it not defunct? Three very important bills in relation to lunacy have been for the last six months under the consideration of the legislature, and no attempt has been made on the part of the Association to summon a meeting of its members to consider their details! Is it not a farce to talk of our having an Association of medical officers of hospitals for the insane, if its members are to be always sleeping at their post, and literally doing nothing to watch the progress of legislation in a department of science, in which it is supposed they are particularly and specially interested? The Association, since its foundation, has published no transactions; neither do we believe any one practical point has been discussed at any one of its meetings. We make these observations in a spirit of great kindness, and solely with the view of urging upon the Association the necessity of constituting itself into a *working body*. These are not the times for apathy and supineness. If we do not do our own work, others will step into our places, and do it for us. How different are the proceedings of the American Association! Its members meet at regular periods, at prearranged localities; papers of great value are read, and afterwards published; and the Association, as a body of intelligent and active psychologists, exercises a great influence over public opinion in America. Let us imitate the example of our transatlantic brethren, put *our* shoulders to the wheel, and do something *collectively* to advance the exalted branch of science which we cultivate.

---

